# Reinforced ProSeal LMA -Revisited

**DOI:** 10.4103/0019-5049.68384

**Published:** 2010

**Authors:** Rajeev Sharma

**Affiliations:** Department of Anaesthesia, ESI Hospital, Rohini, New Delhi, India

Sir,

I read with interest the article titled “Reinforced ProSeal LMA”[1] by Dube *et al*. with interest. The authors have described a use of Foley’s catheter as a means to achieve better seal by PLMA and hypothesized that their technique is superior to the existing design of PLMA, however, there are some reservations against this technique.

First, the authors need to specify in their technique, as to how did they confirm the optimum position of the inflating balloon of Foley’s catheter. It seems they have done it blindly. Blind inflation of the balloon can lead to deformities in the cuff of PLMA and may lead to worsening of the existing seal and possible misplacement of PLMA.

Second, by inflating the balloon beyond the tip of PLMA; the tone of upper oesophageal sphincter could be further decreased leading to increased risk of aspiration.

Third, the aim of provision of drainage tube in PLMA is to provide a channel for gastric suctioning and a vent for air. There are chances that the openings of foley’s catheter abut against the walls of esophagus. In presence of an inflated balloon above it, a regurgitating patient is at risk of developing increased oesophageal pressures and a possibility of oesophageal rupture cannot be ruled out. Further, there is no provision for suctioning the gastric contents using this technique.

Lastly, on what basis the authors have labeled the technique superior and safer is not clear. The article does not mention anything about the measurements of the seal and leak pressures and their comparison without the inflation of balloon. The authors fail to mention, as to how many cases have been done using this technique and need to provide a comprehensive data comparing the positions of cuff, variations in seal and leak pressures apart from relevant data as the cases may be.
